# A longitudinal study on self-rated health changes in disabled older people

**DOI:** 10.3389/fpubh.2024.1372463

**Published:** 2024-05-07

**Authors:** Eunsil Yi, Bogcheon Choi

**Affiliations:** ^1^Jeonbuk Women & Family Foundation, Jeonju, Jeonbuk, Republic of Korea; ^2^Department of Rehabilitation, Jeonju University, Jeonju, Jeonbuk, Republic of Korea

**Keywords:** disabled older people, self-rated health, social exclusion, longitudinal study, multilevel growth model

## Abstract

There is a growing demand for quality healthcare for senior citizens among the disabled older population, considering their rising numbers. This study examines the longitudinal change in the health status of disabled older people and determines its effects on social exclusion and differences based on age at disability onset. The analysis was performed using a multilevel growth model on the health data for disabled older people (≥60 years) derived from the Korea Welfare Panel Study (KWePS). The following findings were observed based on the stated model: (1) The Self-Rated Health (SRH) of disabled older people increased over time, with significant individual differences in the initial status and growth rate; (2) The domains of economic and social network exclusion were associated with changes in the health status of disabled older people; and (3) The longitudinal effects of social exclusion on SRH changes in disabled older people varied according to the age at disability onset. Based on these results, strategies and implications for the development of health-promoting interventions for disabled older people were presented.

## 1 Introduction

Increase in the aging population comes with the problem of their rising disability. Medical and technological advances have prolonged the lifespans of individuals with disabilities. Meanwhile, the prevalence of age-related disabilities has increased alongside the aging population. According to a UN report, 46% of older people (aged 60 years or over) worldwide have disabilities (1). The problems that face disabled older people are increasingly attracting social attention because this population suffers from problems caused by the aging process in addition to their disabilities (2, 3). The biggest difficulty experienced by disabled older people is deteriorating health. They suffer from aging related loss of functions or limitations as well as their disabilities and are at a higher risk of chronic diseases. In addition, people with disabilities may experience an earlier onset of aging compared with people without disabilities (4). The loss of physical functions and the deterioration of health in disabled older people burden them to the extent of their relinquishment of the social activities that used to give them satisfaction, and reduction in their quality of life (5).

Despite the importance of health in the lives of disabled older people, little research has been done on the health problems of this population, primarily because of the notion that damage to health is a static state (6). It is generally assumed that people with disabilities who live with impairments do not need to worry about the consequences of additional deterioration because they have reached their maximum functional capacity by coping with and adapting to their impairments (6, 7). Above all, first, this assumption stems from the belief that people with disabilities comprise the group with the lowest health status because of the general perception that equates disability with ill health, and the health literature in which disability is depicted as the terminal state of chronic disease (8). Second, this assumption comes from the perception that policies related to the health of people with disabilities are hardly likely to obtain the desired effects (9). Disabled older people are stigmatized as a socially vulnerable group and devalued because they have multiple problems of disability and aging (10). While the needs of people with disabilities rapidly increase as they get older, they are often faced with a paradoxical situation of finding that the services provided to them are reduced or even abolished (11). Third, this assumption also arises from the tendency to treat the aging of disabled older people from a pathological perspective and equate aging with disease. This perception leads to the assumption that the decline in physical functions and ill health because of complex chronic diseases or various other disorders among disabled older people are naturally-occurring inevitable phenomena in the aging process.

However, the lives of disabled older people are characterized by social exclusion, and the complex difficulties and alienation experienced by them are the cumulative result of discrimination and social exclusion to which they have been subjected from the age at disability onset to old age (10). Social exclusion is a concept that encompasses not only material deprivation but also poverty-induced alienation and exclusion in the political, sociocultural, and psychological dimensions (12). Thus, social exclusion is manifested in various domains such as dwellings, health, jobs, education, and social networks. In addition, exclusion in each of these domains is interpreted as both the cause and result of exclusion in other domains (12).

An individual’s health status is the result of complex interactions between personal, social, and environmental factors that affect the individual’s daily living. In particular, social exclusion is a social determinant closely associated with health (12, 13). Many studies have demonstrated that social exclusion has significant effects on an individual’s physical and psychosocial health. Per some of the findings of previous studies, it has been observed that older people who are exposed to exclusion from their neighbors are at a higher risk of developing chronic diseases than those who are not (14), those with lower economic status have a higher mortality rate (15), and residents of poor housing environments have poorer physical and mental health (16–18). However, these studies were mostly conducted with older adults or low-income individuals, and rarely with people with disabilities, who are a group exposed to the various risks of social exclusion. This highlights the need to explore their social exclusion and health status.

Additionally, individual experiences of social exclusion summate over time. The older people get, the higher becomes their risk of social exclusion, from which it be-comes increasingly difficult to escape (19). The lives of disabled older people can be understood similarly. The lives of this population are the cumulative results of their lifelong exposure to the conditions of discrimination rather than the possibility of an event occurring at any one moment in old age (20, 21). In particular, health in old age is affected by a wide range of risk factors to which older adults are exposed throughout the human life cycle stages—childhood, adolescence, adulthood, and old age—and their cumulative and latent effects are manifested during their lifetime (22–24).

Therefore, this study aims to determine the trajectory of changes in the health status of disabled older people and assess the effects of social exclusion on health using longitudinal data. An analysis of the time-dependent changes in health and social exclusion in disabled older people will dynamically clarify the situations associated with their health and social exclusion, and contribute to the establishment of effective health-promoting interventions for this population.

## 2 Materials and methods

### 2.1 Participants and materials

Changes in the health of disabled older people were investigated using the supplementary survey data on people with disabilities from the 3rd (2008), 6th (2011), 9th (2014), 12th (2017), and 15th (2020) waves of the Korea Welfare Panel Study (KWePS). The KWePS is a nationwide longitudinal data set that dynamically encapsulates the living conditions and welfare requirements of Koreans, and covers various population groups (25). The study population comprised older people (≥ 60 years at the 3rd wave) with disabilities. In general, old age refers to the second half of life that begins around the age of 65 (3). Among people with disabilities, however, premature aging may start in their 40s or 50s (26). Taking this into account, the UN considers people with disabilities aged 60 or over to belong to the category of older adults (1). Accordingly, people aged 60 years or over were classified as older people in this study. Although 882 people with disabilities are included in the sample, we selected those aged 60 years and over because our target population is disabled older people. A total of 385 disabled older people were included in the analysis.

### 2.2 Measurements of variables

#### 2.2.1 Dependent variable

Self-Rated Health (SRH) was set as the dependent variable in this study. SRH is the measure of an individual’s overall health. Each item is rated on a 5-point scale, with a higher score indicative of a higher perception of overall health. Although higher measurement reliability can be achieved by using a multi-item scale, SRH has been widely used as a single indicator with acceptable reliability to measure health in domestic and foreign studies (27). In particular, Singh-Manoux et al. (28), who conducted a longitudinal analysis using the U.K. Whitehall II study and the French Gazel cohort study samples, emphasized that SRH assessed as a single indicator was “a valid measure of health.” The association between subjective health and objective health is well established. The meta-analysis conducted by Pinquart (29) based on 180 studies revealed that SRH had a strong positive correlation with objective health, such as physical and functional health status. Despite its limitations of being a single-item scale and a subjective measure, the SRH scale is closely associated with major objective health measures, such as mortality and morbidity rate (30) as well as functional limitations and health problems (31). Thus, SRH was deemed appropriate for use as the dependent variable in this study.

#### 2.2.2 Independent variable

Social exclusion was set as the major independent variable in this study, and economic, housing, and social network exclusions were set as the three domains of social exclusion. Economic exclusion was measured by the experience of material deprivation. To determine the experience of material deprivation, the respondents were asked if they had been unable to pay rent, utility bills, tuition fees, or medical bills because of financial constraints. The level of economic exclusion was measured by the level of material deprivation using a scale that comprised 8 binary coded items scored by the assignment of 1 point for yes (experience) and 0 points for no (no experience). A higher total score indicated a higher level of material deprivation, i.e., economic exclusion. Housing exclusion was measured by the level of stability of the residential environment, which was measured using a scale that consisted of 4 binary-coded items on the safety and adequacy of the structure, function, and environment of housing. Each item was rated by the assignment of 0 points for yes and 1 point for no. A higher total score indicated a higher level of housing exclusion. Social network exclusion was measured by a 5-point single-item scale on satisfaction with social relationships that consisted of a single item on a 5-point scale. A higher score (reverse-coded satisfaction score) indicated a higher level of social network exclusion.

#### 2.2.3 Control variable

To determine the changes in the dependent variable, that is, disabled older people’s SRH, factors that may significantly affect SRH were controlled. These factors included disability type, disability severity, age at disability onset, household income at disability onset, and sex. Disability type was categorized as physical (=1) and non-physical (=0) disabilities, and disability severity as severe (=1) and mild (=0). To understand and investigate disabled older people’s SRH, it is necessary to recognize its temporal dimension, that is, not only the general process of aging but also the age at disability onset and duration of living with the disabilities. Age at disability onset and duration provide information related to therapeutic opportunities or treatment experiences (3). Therefore, the age at disability onset was included as the control variable and used as the reference value for the division of the participants into the “aging with disability” (AWD) and “disability with aging” (DWA) groups. In general, older people who are disabled at birth or in childhood are considered to “age with disability,” and those free of disability until late life are considered to experience “disability with aging” (3, 32). In this study, AWD and DWA denote people whose age at disability onset was before, and at/after the age of 60, respectively.

### 2.3 Analysis methods

Stata 16.0 was used for data processing and statistical analysis. The participants’ sociodemographic characteristics and the characteristics of major variables were analyzed using descriptive statistics and frequency analysis. A multilevel growth model was used to determine the level of social exclusion and SRH in disabled older people, and assess their correlations with the factors associated with individual differences. Multilevel growth model is used because this method has following advantages. It can assess the changing trajectories and patterns of variables and consider the individual differences that are observed in the process of change. In addition, this method has ability to incorporate time-varying predictors, handle dependence among repeated observations in a very flexible manner, and to provide accurate estimates with missing data under fairly unrestrictive missing data assumptions (33). Older adults with disabilities are at a higher risk of attrition during repeated measurement processes. Treating participants with insufficient measurement occasions as missing data can reduce the precision of the analysis. Multilevel growth model analysis utilizes Maximum Likelihood Estimation, which is suitable for analyzing unbalanced data and accommodates cases with varying numbers of measurements. This method, therefore, allows for valid analysis without data loss (34).

In the first stage of the model analysis, the growth equation of the dependent variable (SRH) and the time-dependent independent variable (social exclusion) was estimated using the unconditional model. In the second stage, disability-related covariates and social exclusion (independent variable) were entered as factors, which can explain individual differences in change patterns, to determine their impacts on changes in SRH in disabled older people. The equations of the conditional model for the estimation of the changing trend of SRH in disabled older people are as follows:

Level-1:


$$\begin{array}{l} Y_{ti}=\pi_{0i}+\pi_{1i}{\left(\mathrm{time}\right)}_{ti}+\pi_{2i}{\left(EE\right)}_{\left(t-1\right)i}+\pi_{3i}{\left(\mathrm{HE}\right)}_{\left(t-1\right)i}+\\ \;\pi_{4i}{\left(SNE\right)}_{\left(t-1\right)i}+e_{ti},e_{ti}\sim N\left(0,\sigma^{2}\right) \end{array}$$

Level-2:


$$\begin{array}{l} \pi_{0i}=\beta_{00}+\beta_{01}X1_{1i}+\beta_{02}X2_{2i}+\beta_{03}X3_{3i}+\\ \;\beta_{04}X4_{4i}+\beta_{05}X5_{5i}+r_{0i} \end{array}$$

$$\pi_{1i}=\beta_{10}+\beta_{11}X1_{1i}+\beta_{12}X2_{2i}+\beta_{13}X3_{3i}+r_{1i}$$

$$\pi_{2i}=\beta_{20}$$

$$\pi_{3i}=\beta_{30}$$

$$\pi_{4i}=\beta_{40}$$

*(EE: economic exclusion, HE: housing exclusion, SNE: social network exclusion, X1: age at disability onset, X2: household income at disability onset, X3: disability severity, X4: disability type, X5: sex).*

## 3 Results

### 3.1 Participants’ sociodemographic characteristics

The demographic characteristics of the participants are outlined in Table 1: males outnumbered females (58.44 to 41.56%); the 65–69 age group accounted for 36.61% of the total disabled aging population, followed by the 60–64 (24.42%), 70–74 (21.82%), and ≥ 75 (17.16%) age groups; physical disabilities dominated over non-physical ones (61.82 to 38.18%); severe disabilities outnumbered mild disabilities (55.58% vs. 44.42%). Disability onset age distribution was 10.9% for adolescents (≤ 18 years), 54.6% for younger adults (19–59), and 34.5% for older adults (≥ 60). With regard to household income at disability onset, 14.29% of the households were in the “very low” category, 50.39% in the “low,” 32.73% in the “middle,” and 2.6% in the “high” categories, which demonstrated that a majority of the households of disabled older people belonged to the low-income group.

**Table 1:** Sociodemographic characteristics.

Variables	Category	Frequency (*n*)	Ratio (%)
Sex	Female	160	41.56
	Male	225	58.44
Age	60*–*64	94	24.42
	65*–*69	141	36.61
	70*–*74	84	21.82
	≥75	66	17.16
Disability type	Physical disabilities	238	61.82
	Non-physical disabilities	147	38.18
Disability severity	Severe	214	55.58
	Mild	171	44.42
Age at disability onset	0–18	42	10.9
	19–59	210	54.6
	≥60	133	34.5
Household income at disability onset	Very low	55	14.29
	Low	194	50.39
	Middle	126	32.73
	High	10	2.6

### 3.2 Characteristics of major variables

The descriptive statistics of social exclusion (independent variable) and SRH (dependent variable) are presented in Table 2, which demonstrates the longitudinal changes in social exclusion and SRH in disabled older people during the period covering the 3rd to 16th waves of the KWePS. It was found that while SRH increased over time, economic and social network exclusions decreased over time.

**Table 2:** Descriptive statistics of variables.

Waves	Self-rated health		Economic exclusion		Housing exclusion		Social network exclusion	
	Mean	SD	Mean	SD	Mean	SD	Mean	SD
3rd	2.14	0.89	0.16	0.48	2.52	0.84	2.74	1.03
6th	2.34	0.86	0.13	0.39	2.70	0.65	2.62	0.83
9th	2.30	0.83	0.09	0.36	2.67	0.68	2.58	0.75
12th	2.38	0.81	0.04	0.23	2.76	0.58	2.59	0.75
15th	2.44	0.81	0.02	0.17	3.43	0.96	2.65	0.78

### 3.3 Analysis of the study model

#### 3.3.1 Unconditional model

An unconditional model was applied to estimate the growth equation. Table 3 demonstrates that the variance estimate of SRH in disabled older people was 0.527 and 0.224 at the levels 1 and 2 measurements, respectively. The ICC value of SRH was 0.298, which suggests that the inter-individual effect explained 29.8% of the total change in SRH. As for economic exclusion, the variance estimate was 0.132 at the level 1 measurement, and 0.039 at the level 2 measurement. With an ICC value of 0.226, the inter-individual effect explained 22.6% of the total change in economic exclusion. The level 1 and level 2 variance estimates of housing exclusion were 0.644 and 0.157, respectively; with an ICC value of 0.196, the inter-individual effect explained 19.6% of the total change in housing exclusion. Lastly, the individual effect rate of social network exclusion was 9.3%, with an ICC value of 0.093.

**Table 3:** Null model analysis results.

	Self-rated health	Economic exclusion	Housing exclusion	Social network exclusion
Cons	2.199	0.127	2.848	2.674
Level 1	0.527	0.132	0.644	0.657
Level 2	0.224	0.039	0.157	0.067
ICC	0.298	0.226	0.196	0.093

Following this, time covariates and random effect were added and analyzed to examine the longitudinal change of SRH. Table 4 presents the results of the linear model analysis. It was observed that SRH increased at significant growth rates over time. The mean initial status and growth rate of SRH were 2.222 (*p* < 0.000) and 0.023 (*p* < 0.001), respectively. The growth rates were statistically significant, which indicates that disabled older people’s SRH constantly rose for the observation period of 12 years. The random effect of initial status and growth rate was statically significant, which indicates that individual differences existed in disabled older people’s initial status and growth rate. In addition, the initial status and growth rate of SRH demonstrated a negative correlation coefficient (−0.032): those with a higher SRH compared with the initial measurement were found to change more slowly over time than those who had a lower SRH.

**Table 4:** Linear model analysis results.

	Coef.	SE
$\beta_{00}$	2.222***	0.027
$\beta_{10}$	0.023**	0.008
$\tau_{00}$	0.283	0.035
$\tau_{11}$	0.009	0.003
$\tau_{10}$	−0.032	0.009

* *p* < 0.05, ** *p* < 0.01, *** *p* < 0.001.

#### 3.3.2 Conditional model

In the previous section, SRH demonstrated a linearly increasing pattern over time in the unconditional model, along with individual differences. In this regard, the conditional model was applied using the covariates that can explain individual differences as input variables. Table 5 outlines the analysis results for the conditional growth model.

**Table 5:** Conditional model analysis results.

Variables	Model 1		Model 2		Model 3	
Fixed effect	Coef.	SE	Coef.	SE	Coef.	SE
Initial status intercept	2.301***	0.029	2.437***	0.055	2.406***	0.058
Sex			0.031	0.042	0.064	0.040
Disability type			−0.031	0.043	−0.017	0.040
Disability severity			−0.150***	0.041	−0.122*	0.063
Age at disability onset			−0.004***	0.001	−0.007***	0.002
Household income at disability onset			0.066*	0.029	0.067	0.043
Growth rate intercept	0.025**	0.009	0.025**	0.012	0.030	0.022
Economic exclusion	−0.952**	0.036	−0.049*	0.033	−0.090*	0.040
Housing exclusion	0.006	0.018	0.005	0.020	0.005	0.020
Social network exclusion	−0.094**	0.017	−0.076**	0.019	−0.095***	0.019
Time*Severity of disabilities					−0.002	0.025
Time*Household income at disability onset					−0.003	0.014
Time* Age at disability onset					0.001**	0.000
Random effect	Estimate	SE	Estimate	SE	Estimate	SE
Intercept	0.237	0.035	0.207	0.040	0.186	0.035
Time (slope)	0.009	0.003	0.013	0.004	0.008	0.004
Cov (time, _cons)	−0.031	0.01	−0.035	0.012	−0.027	0.010
Level 1-error	0.498	0.017	0.497	0.019	0.497	0.018

* *p* < 0.05, ** *p* < 0.01, *** *p* < 0.001.

In model 1, time-variant covariates were entered for the analysis of within-individual variations. An analysis revealed that disabled older people’s SRH was influenced by the measurement time and individual characteristics. Statistically significant individual differences were found in the initial status and growth rate of the random effect. Disabled older people’s mean initial status and SRH growth rate were calculated at 2.301 and 0.025, respectively, with statistical significance. An analysis of the inter-variable causal relations led to the finding that economic and social network exclusions had a significant effect on the longitudinal changes in SRH and that the effect of housing exclusion was not statistically significant.

In Model 2, time-invariant covariates were entered for analysis at level 2. The analysis confirmed statistically significant individual differences in the initial status and growth rate of random effect (Log likelihood = −2310.9419, chi2(16)=309.33, (*p* < 0.000)). The variances of SRH at the measurement time point (level 1) and the initial status (level 2) were calculated at 0.497, and 0.207, respectively, and disability severity, age at disability onset, and household income at disability onset were found to significantly affect disabled older people’s initial SRH status. That is, a higher initial SRH status was associated with mild disability, earlier disability onset, and higher household income at disability onset. Among the three domains of social exclusion, the initial status of social network exclusion was found to influence the initial SRH status in the independent variable.

Finally, in Model 3, the interaction terms of the time covariates of level 1 and disability severity, age at disability onset, and household income at disability onset of level 2 were additionally entered as input variables to determine the effects of the time-invariant covariate on the changing trend of SRH over time, whereby grand-mean centering was applied to level 2 covariates. The analysis of the final SRH model revealed that statistically significant individual differences existed in the initial status and growth rate. Disabled older people’s mean initial status and growth rate of SRH were 2.406 (*p* < 0.001) and 0.03 (*p* < 0.01), respectively, with statistical significance. The initial SRH was found to be influenced by disability severity (β = −0.122, *p* < 0.05) and age at disability onset (β = −0.007, *p* < 0.000). The SRH growth rate was influenced by economic exclusion (β = −0.090, *p* < 0.05) and social network exclusion (β = −0.095, *p* < 0.000). As for the interaction effect with time covariates, the effects of disability severity and household income at disability onset were not found to be significant, while that of the age at disability was significant.

**Figure 1:**
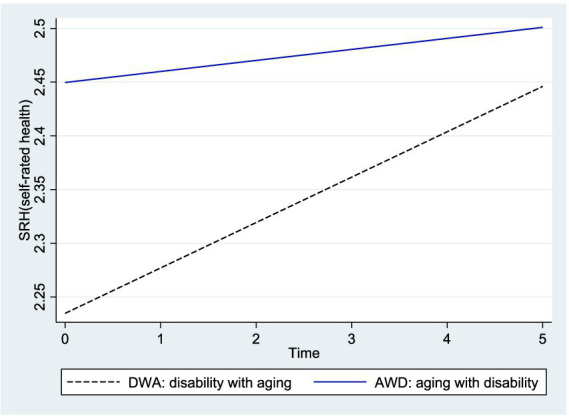
Interaction effect between time and the age at disability onset.

The graphs in Figure 1 illustrate the interaction effect (moderating effect) between time and age at disability onset in the DWA and AWD groups. SRH increases over time in both groups, but the DWA group has a steeper slope of change than the AWD; the health disparities between them increasingly narrow over time.

## 4 Discussion

This study aimed to examine how disabled older people’s SRH changes over time, analyze how their social exclusion influences the changes in their SRH, and assess whether there are individual differences based on the age at disability onset. The multilevel growth model was used as the analysis method, and data from the 3rd to 15th waves of the KWePS were utilized. This section discusses the details of the results of this study.

First, the results revealed that disabled older people’s SRH levels improved over time. Thus, it may be considered that disabled older people evaluate their health more positively as they get older. This finding is not consistent with those of previous research that presupposed the health status of people with disabilities as an invariable static condition (6) or reported that their health status declined with time because the health vulnerability of disabled older people is a loss inherent in the aging process (35). The results of this study can be interpreted from two perspectives, the first being the maturation effect of the samples (36). In longitudinal studies, dependent variables may be influenced by the naturally changing physical and psychological characteristics of the participants. Therefore, the disabled older people who participated in this study may have naturally accepted their disabilities and impairments over time during the 12-year observation period and gained the awareness that disability is not a result of health deterioration. The other perspective is that this study’s results reflect the phenomenon of cumulative equalities. Given the fact that people who are frequently exposed to adverse life events early in life are more likely to die early deaths, and that healthy people generally live longer, different life experiences can lead to changes in the composition of the older adult population. Ferraro et al. (37) noted that caution is warranted in the interpretation of this reversal phenomenon of population composition, lest inequality should reduce in old age.

Second, social exclusion was found to have a significant effect on the initial status and growth rate of SRH in disabled older people. Kemp et al. (38) observed that the psychological health of people with polio who reached old age significantly deteriorated only when they experienced changes in social relationships or lost social cohesion. That is, exclusion from social relationships was found to affect health more seriously than the severity or duration of disability. The results of this study also support this finding. Therefore, while it is important to consider the characteristics or severity of disability, it is necessary to focus more on the level and extent of social exclusion in the planning of health-promotion interventions for disabled older people. Until now, health has been treated as an individual responsibility from a medical point of view. However, the results of this study, which revealed the longitudinal relationship between social exclusion and SRH in disabled older people, identify the need to approach health from a community perspective. Economic exclusion and social network exclusion were found to have lasting, significant effects on disabled older people’s SRH. The World Health Organization (WHO) holds a holistic view of health by defining health as “a state of complete physical, mental, and social well-being and not merely the absence of disease or infirmity” (12). From this perspective, a desirable method by which to help disabled older people maintain stable health would be to ensure that they actively participate in, and are not excluded from, various life areas.

To this end, it is necessary to expand the social participation of disabled older people and endeavor to maintain their existing social network, which is what this population desires most. This study reaffirms that social network exclusion is an important predictor of SRH among disabled older people. It highlights the need to provide them with interventions designed to promote social participation and maintain meaningful social networks. For example, the provision of various programs within the community, including recreational activities, educational programs, clubs, and other community activities, can significantly enhance the formation of social networks. It is also worth noting that older people with disabilities that are incurred at an early age are more easily exposed to poverty and deprivation. This is because disabilities incurred in the early years of life can lead to exclusion from education and the labor market, and ultimately, excessive economic exclusion in the later years of life because of the cumulative burden of additional costs incurred for their coping with their disabilities. Therefore, it is necessary to expand the National Pension Service (NPS) and adopt an income security policy to protect people with disabilities from suffering from economic exclusion in old age.

Third, the longitudinal effects of social exclusion on the changes of SRH in disabled older people were found to vary according to the age at disability onset. This denotes that differentiated interventions should be provided based on the age at disability onset to mitigate health inequalities among disabled older people. The health disparities between the AWD and DWA groups were most pronounced in the early stage of disability and became narrower over time. This can be interpreted as the difference that occurred before they reached old age. Therefore, for disabled older people to live to a healthier old age, rehabilitation and health management should be implemented in the early stages of disability.

This study is significant in that it examines the longitudinal change of SRH and health disparities in disabled older people using longitudinal data. However, since it used secondary data, it had a limited set of variables. Therefore, the results of this study should be interpreted with caution. In this study, social exclusion was examined in the domains of economic, housing, and social network exclusions, which are individual and microscopic aspects of social exclusion. The community environment is an important area of social exclusion from a macroscopic perspective (39); however, this could not be considered for analysis in this study because of data limitations, which must be addressed in future research.

## 5 Conclusion

Along with the rapidly increasing aging population, the number of older people with disabilities has also surged in recent years, which has led to an increase in research on disability and aging, and the health inequalities between people with disabilities and the general population. However, extant studies have focused on the gaps between people with disabilities and those without, and little research attention has been paid to the examination of the differences among people with disabilities. Additionally, most studies have used cross-sectional data, and not considered the changes in SRH over time.

In this study, longitudinal data were used to examine changes in the SRH among disabled older people over time. Specifically, the effects of social exclusion were assessed with a focus on the differences based on the age at disability onset. The results of the study can be summarized as follows. First, the SRH of disabled older people improved progressively, which may be ascribed to the psychological maturation effect that has positively affected the participants’ SRH over time. Second, economic exclusion and social network exclusion were identified as risk factors for the health of disabled older people. Therefore, it is necessary to develop intervention programs as well as services to expand social participation opportunities for disabled older people and support their maintenance of meaningful social networks. Additionally, various income security policies should be established and implemented to mitigate the economic exclusion experienced by disabled older people. Third, significant differences in SRH were observed between the AWD and DWA groups. These results are expected to serve as a basis for the understanding of the differences in health characteristics between the AWD and DWA groups and the establishment of tailored intervention strategies in a world where the number of disabled older people is gradually increasing.

## Data availability statement

Publicly available datasets were analyzed in this study. This data can be found here: http://www.koweps.re.kr.

## Ethics statement

Ethical approval was not required for the study involving humans in accordance with the local legislation and institutional requirements. Written informed consent to participate in this study was not required from the participants or the participants’ legal guardians/next of kin in accordance with the national legislation and the institutional requirements.

## Author contributions

EY: Conceptualization, Formal analysis, Methodology, Writing – original draft. BC: Formal analysis, Methodology, Writing – review & editing.
